# Telehealth Availability for Mental Health Care During and After the COVID-19 Public Health Emergency

**DOI:** 10.1001/jamanetworkopen.2024.20853

**Published:** 2024-07-10

**Authors:** Ryan K. McBain, Megan S. Schuler, Joshua Breslau, Aaron Kofner, Lulu Wang, Jonathan H. Cantor

**Affiliations:** 1 Division of Healthcare Delivery, RAND, Washington, DC; 2 Department of Medicine, Brigham and Women’s Hospital, Boston, Massachusetts; 3 Division of Healthcare Delivery, RAND, Pittsburgh, Pennsylvania; 4 School of Medicine, Georgetown University, Washington, DC; 5 Division of Healthcare Delivery, RAND, Santa Monica, California

## Abstract

**Importance:**

Telehealth services expanded rapidly during the COVID-19 public health emergency (PHE).

**Objective:**

To evaluate changes in availability of telehealth services at outpatient mental health treatment facilities (MHTFs) throughout the US during and after the COVID-19 PHE.

**Design, Setting, and Participants:**

In this cohort study, callers posing as prospective clients contacted a random sample of 1404 MHTFs drawn from the Substance Abuse and Mental Health Services Administration’s Behavioral Health Treatment Locator from December 2022 to March 2023 (wave 1 [W1]; during PHE). From September to November 2023 (wave 2 [W2]; after PHE), callers recontacted W1 participants. Analyses were conducted in January 2024.

**Main Outcomes and Measures:**

Callers inquired whether MHTFs offered telehealth (yes vs no), and, if yes, whether they offered (1) audio-only telehealth (vs audio and video); (2) telehealth for therapy, medication management, and/or diagnostic services; and (3) telehealth for comorbid alcohol use disorder (AUD). Sustainers (offered telehealth in both waves), late adopters (did not offer telehealth in W1 but did in W2), nonadopters (did not offer telehealth in W1 or W2), and discontinuers (offered telehealth in W1 but not W2) were all compared.

**Results:**

During W2, 1001 MHTFs (86.1%) were successfully recontacted. A total of 713 (71.2%) were located in a metropolitan county, 151 (15.1%) were publicly operated, and 935 (93.4%) accepted Medicaid as payment. The percentage offering telehealth declined from 799 (81.6%) to 765 (79.0%) (odds ratio [OR], 0.84; 95% CI, 0.72-1.00; *P* < .05). Among MHTFs offering telehealth, a smaller percentage in W2 offered audio-only telehealth (369 [49.3%] vs 244 [34.1%]; OR, 0.53; 95% CI, 0.44-0.64; *P* < .001) and telehealth for comorbid AUD (559 [76.3%] vs 457 [66.5%]; OR, 0.62; 95% CI, 0.50-0.76; *P* < .001) compared with W1. In W2, MHTFs were more likely to report telehealth was only available under certain conditions for therapy (141 facilities [18.0%] vs 276 [36.4%]; OR, 2.62; 95% CI, 1.10-3.26; *P* < .001) and medication management (216 facilities [28.0%] vs 304 [41.3%]; OR, 1.81; 95% CI, 1.48-2.21; *P* < .001). A total of 684 MHTFs (72.0%) constituted sustainers, 94 (9.9%) were discontinuers, 106 (11.2%) were nonadopters, and 66 (7.0%) were late adopters. Compared with sustainers, discontinuers were less likely to be private for-profit (adjusted OR [aOR], 0.28; 95% CI, 0.11-0.68) or private not-for-profit (aOR, 0.26; 95% CI, 0.14-0.48) after adjustment for facility and area characteristics.

**Conclusions and Relevance:**

Based on this longitudinal cohort study of 1001 MHTFs, telehealth availability has declined since the PHE end with respect to scope and modality of services, suggesting targeted policies may be necessary to sustain telehealth access.

## Key Points

**Question:**

Has availability of telehealth for mental health care declined since the end of the COVID-19 public health emergency (PHE)?

**Findings:**

This cohort study of 1001 mental health treatment facilities (MHTFs) found a small decline in MHTFs offering telehealth subsequent to the end of the PHE and found larger declines in availability of audio-only telehealth services and care for comorbid alcohol use disorder. MHTFs that stopped offering telehealth were more likely to be public facilities.

**Meaning:**

These findings suggest the end of the PHE was associated with declines in telehealth availability for individuals with mental health conditions.

## Teaser

This cohort study evaluates changes in availability of telehealth services at outpatient mental health treatment facilities throughout the US during and after the COVID-19 public health emergency.

## Introduction

Over the course of the COVID-19 pandemic, telehealth availability expanded rapidly.^1^ This change was marked and persistent for mental health care, for which most services do not require in-person physical examinations or diagnostic tests.^2^ State and federal policies enacted during the pandemic promoted the telehealth transition by altering Medicare to reimburse for telehealth services^3^ and state Medicaid agencies to approve reimbursement for audio-only telehealth, for example.^4^

On May 11, 2023, the Biden Administration declared the end of the COVID-19 public health emergency (PHE).^5^ Correspondingly, a range of telehealth regulations tethered to the PHE also expired on this date.^6^ Others, particularly those associated with Centers for Medicare and Medicaid Services (CMS), are set to expire at the end of 2024.^7^ In this study, we conducted a national secret shopper analysis, comparing mental health treatment facilities’ (MHTF) responses about availability of telehealth before vs after the end of the PHE.

## Methods

We contacted MHTFs in a 3-month period between December 2022 and March 2023 and again 6 months later between September and November 2023. Callers posed as adult clients inquiring about availability and features of telehealth services. Data were combined with aggregate facility- and county-level characteristics for analysis.

This cohort study was approved by the RAND Human Subjects Protection Committee. Informed consent was waived because the study was deemed not to constitute human participants research. This report follows the Strengthening the Reporting of Observational Studies in Epidemiology (STROBE) reporting guideline for observational studies.

### Sampling

The sampling frame comprised outpatient MHTFs throughout the US, recorded in the Substance Abuse and Mental Health Services Administration’s (SAMHSA) Behavioral Health Treatment Service Locator (BHTSL) on August 22, 2022,^8^ which represents a national inventory of psychiatric facilities. The BHTSL does not include private practices. The BHTSL is updated on a monthly basis and includes facility characteristics such as types of services offered, insurances accepted, and public vs private ownership.^9^ We abstracted facility address and phone number for contact purposes.

Of 9568 outpatient MHTFs within the BHTSL on August 22, 2022, we randomly selected 25% (1938) for contact in wave 1, of which we successfully contacted 1404 (72.5%) between December 2022 and March 2023. In wave 2 (September to November 2023), we recontacted 1163 that we successfully contacted in wave 1, achieving a wave 2 sample of 1001 facilities (86.1%). In both waves, facility addresses were linked to county-level information using the Health Resources and Services Administration’s Area Health Resource Files,^10^ including county metropolitan status (metropolitan vs nonmetropolitan), percentage of residents who are Black, percentage of residents who are Hispanic, and median household income.

### Procedures

Trained callers read from a standardized script.^11,12^ Callers posed as prospective clients with 1 of 3 clinical conditions—randomly assigned to callers at the facility-level—for which they were seeking services: major depressive disorder, generalized anxiety disorder, or schizophrenia. Callers inquired about specific aspects of telehealth availability, as described in the next section. Callers documented facility responses in Qualtrics XM (Qualtrics).

### Outcomes

Our primary outcome was a binary measure of whether the facility was currently offering telehealth (yes vs no). Among facilities reporting they offered telehealth, we also inquired about the telehealth modalities offered (audio-only vs video requirement) and whether facilities offered telehealth services for individuals with a mental health condition and comorbid alcohol use disorder (AUD; yes vs no). Lastly, we assessed whether facilities offering telehealth provided 3 types of services: telehealth-based psychotherapy, telehealth-based medication management, and telehealth-based diagnostic services (yes vs it depends vs no). The full protocol can be found in the eAppendix in Supplement 1.

### Statistical Analysis

To examine nonresponse bias, we compared responders in waves 1 and 2 with the full sampling frame, as well as responders in waves 1 and 2 vs 1 only. We determined whether the differences between these groups was statistically significant using a χ^2^ test. Among those in the analytic sample (1001 MHTFs), we reported descriptive statistics comparing survey responses for wave 1 vs wave 2. For each survey response item, we tested for significant change across waves using univariate logistic regression models, with SEs clustered at the facility level.

For the primary outcome (any telehealth offered), we categorized facilities into 4 groups: sustainers who responded yes in both waves; nonadopters who responded no in both waves; late adopters who responded no in wave 1 and yes in wave 2; and discontinuers who responded yes in wave 1 and no in wave 2. We then conducted fixed-effects multinomial regression analysis to examine the associations between group membership and both facility-level characteristics (public vs private and accepting Medicaid vs not) and county-level characteristics (metropolitan vs nonmetropolitan, percentage of residents who are Hispanic, percentage of residents who are Black, and median household income). SEs were clustered at the state level.

All analyses were conducted in Stata version 17.0 (StataCorp). Statistical tests were 2-sided, using an α threshold of .05. Analyses were conducted in January 2024.

## Results

### Study Overview

Within the analytic sample, 713 facilities (71.2%) were located in metropolitan counties compared with 288 (28.8%) in nonmetropolitan counties. Additionally, 638 (63.7%) were private not-for-profit, 212 (21.2%) were private for-profit, and 151 (15.1%) were public. A total of 935 (93.4%) accepted Medicaid, while the remainder did not.

Compared with the sampling frame, facilities in the analytic sample were more likely to be publicly owned (151 [15.1%] vs 47 [11.7%]; χ^2^ = 6.21; *P* = .045), accept Medicaid as a form of payment (935 [93.4%] vs 355 [88.1%]; χ^2^ = 10.89; *P* = .001), and be in a county where the median household income was below the national median (330 [33.0%] vs 109 [27.1%]; χ^2^ = 4.69; *P* = .03). Facilities in the analytic sample were also more likely to be located in counties in the lowest quartile of non-Hispanic and African American residents (458 [45.8%] vs 168 [41.7%]).

As shown in Table 1, overall availability of telehealth declined slightly—from 799 facilities (81.6%) to 765 (79.0%)—between waves (odds ratio [OR], 0.84; 95% CI, 0.72-1.00; *P* = .046). By contrast, availability of audio-only telehealth declined from 369 facilities (49.3%) to 244 (34.1%) (OR, 0.53; 95% CI, 0.44-0.64; *P* < .001) and availability of telehealth for comorbid mental health and AUD declined from 559 facilities (76.3%) to 457 (66.5%) (OR, 0.62; 95% CI, 0.50-0.76; *P* < .001). With respect to telehealth for psychotherapy, there was a significant decline in the percentage reporting yes (OR, 0.39; 95% CI, 0.31-0.48) and significant growth in the portion reporting it depends (OR, 2.62; 95% CI, 2.10-3.26). The same was observed for medication management; there was a significant decline in the percentage reporting yes (OR, 0.59; 95% CI, 0.49-0.71) and significant growth in the portion reporting it depends (OR, 1.81; 95% CI, 1.48-2.21).

**Table 1.** Characteristics of Telehealth Services at Mental Health Treatment Facilities During and After Public Health Emergency (PHE)

Characteristic	Respondents, No. (%)		*P *value^b^
	Wave 1: during PHE^a^	Wave 2: after PHE	
Any telehealth?			
Yes	799 (81.6)	765 (79.0)	.046
No	180 (18.4)	204 (21.1)	
Telehealth modality			
Audio-only	369 (49.3)	244 (34.1)	<.001
Both audio and video formats	380 (50.7)	471 (65.9)	
Comorbid mental health/SUD telehealth?			
Yes	559 (76.3)	457 (66.5)	<.001
No	174 (23.7)	230 (33.5)	
Telehealth-based psychotherapy?			
Yes	625 (79.6)	457 (60.3)	<.001
It depends	141 (18.0)	276 (36.4)	<.001
No	19 (2.4)	25 (3.3)	.26
Telehealth-based medication management?			
Yes	367 (47.5)	257 (34.9)	<.001
It depends	216 (28.0)	304 (41.3)	<.001
No	189 (24.5)	175 (23.8)	.07
Telehealth-based diagnostic services?			
Yes	368 (49.4)	318 (45.2)	.08
It depends	153 (20.5)	145 (20.6)	.99
No	224 (30.1)	240 (34.1)	.07

Abbreviation: SUD, substance use disorder.

^a^ PHE expired on May 11, 2023.

^b^ *P* values derived from univariate logistic regression models with SEs clustered at the facility level.

### Trajectories of Telehealth Availability

Overall, 674 facilities (72.0%) were in the sustainer class, 106 (11.2%) were in the nonadopter class, 66 (7.0%) were in the late adopter class, and 94 were (9.9%) in the discontinuer class. The Figure depicts the geographic distribution. We note that discontinuers and nonadopters generally appear to be concentrated in the southeastern US.

**Figure.**
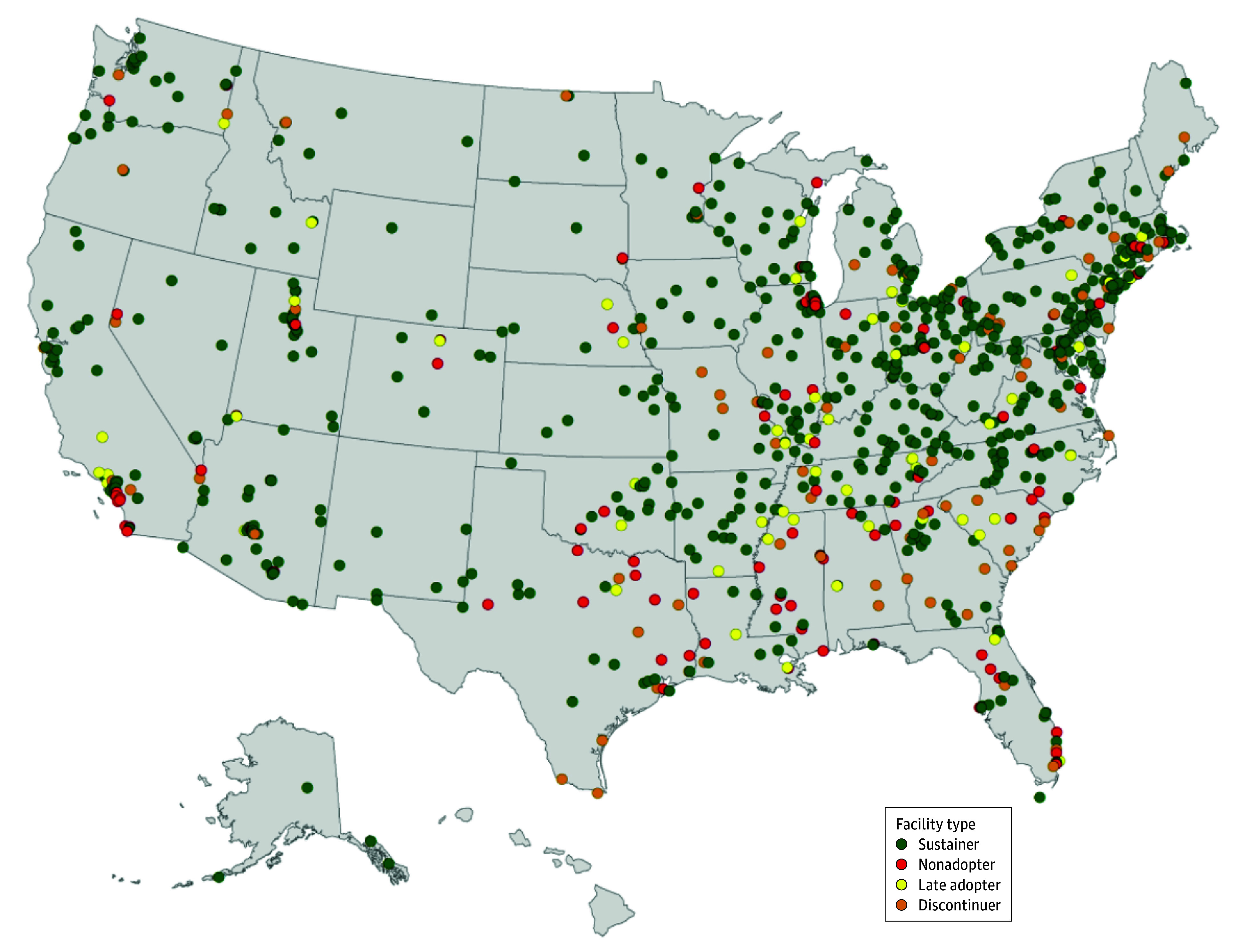
Telehealth Availability Status at Mental Health Treatment Facilities During and After COVID-19 Public Health Emergency Facilities were classified based on their wave 1 and 2 responses to the question: “Do you provide any telehealth services?” Sustainers were those that offered telehealth during and after the end of the public health emergency (PHE). Discontinuers were those that offered telehealth during but not after the end of the PHE. Nonadopters were those that did not offer telehealth during or after the end of the PHE. Late adopters were those that started offering telehealth after the end of the PHE.

As shown in Table 2, results suggested MHTF ownership was significantly associated with class membership. Relative to public MHTFs, private for-profit MHTFs had 72.0% lower odds of being a discontinuer (adjusted OR [aOR], 0.28; 95% CI, 0.11-0.69) and 53.5% lower odds of being a nonadopter (aOR, 0.47; 95% CI, 0.22-0.99) compared with sustainers. Similarly, relative to public MHTFs, private not-for-profit MHTFs had 73.8% lower odds of being a discontinuer (aOR, 0.26; 95% CI, 0.14-0.48) and 73.7% lower odds of being a nonadopter (aOR, 0.26; 95% CI, 0.15-0.47).

**Table 2.** Facility- and County-Level Differences in Trajectories of Telehealth Availability by Class

Characteristic	aOR (95% CI)^a^		
	Discontinuers	Nonadopters	Adopters
Facility type			
Public	1 [Reference]	1 [Reference]	1 [Reference]
Private for-profit	0.28 (0.11-0.68)^b^	0.47 (0.22-0.99)^c^	0.57 (0.24-1.38)
Private not-for-profit	0.26 (0.14-0.48)^d^	0.26 (0.15-0.47)^d^	0.63 (0.30-1.35)
Accepts Medicaid	0.84 (0.28-2.53)	0.33 (0.16-0.69)^b^	0.82 (0.21-3.11)
Metropolitan	1.56 (0.85-2.89)	1.64 (0.93-2.90)	1.40 (0.75-2.61)
Percentage Hispanic			
Lowest quartile	1 [Reference]	1 [Reference]	1 [Reference]
Second quartile	0.76 (0.38-1.51)	0.82 (0.38-1.78)	1.01 (0.57-1.80)
Third quartile	1.01 (0.51-2.03)	1.39 (0.64-3.01)	0.80 (0.35-1.85)
Highest quartile	0.94 (0.41-2.19)	1.97 (1.07-3.64)^c^	0.73 (0.33-1.60)
Percentage Black			
Lowest quartile	1 [Reference]	1 [Reference]	1 [Reference]
Second quartile	1.47 (0.81-2.68)	1.20 (0.44-3.28)	1.42 (0.74-2.69)
Third quartile	1.15 (0.57-2.32)	1.44 (0.66-3.11)	0.73 (0.26-2.10)
Highest quartile	1.40 (0.73-2.67)	1.59 (0.66-3.84)	1.71 (0.82-3.59)
Median income			
Below median	1 [Reference]	1 [Reference]	1 [Reference]
Above median	0.80 (0.47-1.39)	0.88 (0.58-1.33)	0.84 (0.52-1.37)

Abbreviation: aOR, adjusted odds ratio.

^a^ For all measures, reference group for outcome is sustainers category (684 facilities). Sustainers were those that offered telehealth during and after the end of the public health emergency (PHE). Discontinuers were those that offered telehealth during but not after the end of the PHE. Nonadopters were those that did not offer telehealth during or after the end of the PHE. Adopters were those that started offering telehealth after the end of the PHE.

^b^ *P* < .01.

^c^ *P* < .05.

^d^ *P* < .001.

Among public MHTFs, 52 (18.4%) were nonadopters, 58 (20.6%) were discontinuers, and 150 (53.2%) were sustainers. In contrast, private facilities were more likely to be sustainers (292 for-profits [69.9%]; 926 not-for-profits [77.2%]) and less likely to be nonadopters (66 for-profits [15.8%]; 34 not-for-profits [8.1%]) or discontinuers (94 for-profits [7.8%]; 96 not-for-profits [8.0%]). Additionally, MHTFs that accepted Medicaid had 33.5% (66.5%) lower odds of being a nonadopter (aOR, 0.34; 95% CI, 0.16-0.69) relative to MHTFs that did not accept Medicaid. MHTFs in communities with the highest quartile of proportion of Hispanic residents were more likely to be nonadopters than MHTFs in communities in the lowest quartile of proportion of Hispanic residents (aOR, 1.97; 95% CI, 1.07-3.64).

## Discussion

Our results suggest telehealth coverage for mental health services have contracted slightly since the end of the PHE, coincident with changes (and anticipated changes) in public and commercial payer reimbursement policies following the end of the COVID-19 PHE.^5^ While only 2.6% fewer MHTFs were offering telehealth after the PHE, there was lower adoption and higher discontinuation of telehealth in public than in private MHTFs, suggesting that publicly owned MHTFs may face additional barriers to telehealth promotion. This finding is consistent with recent studies in the literature.^13,14^

In addition, we found that MHTFs offering telehealth at wave 2 were providing more limited services—for example, more frequently requiring video-based telehealth, declining services for comorbid AUD, and placing limitations on availability of telehealth services for psychotherapy and medication management compared with wave 1. One potential explanation for this is that the end of the PHE may have had a chilling effect on clinician behavior; during the COVID-19 pandemic, HIPAA and regulatory agencies offered broad discretion to clinicians to encourage telehealth utilization for nonemergency services.^15^ By contrast, state and federal guidance has become more stringent following the conclusion of the PHE, including by CMS.^7^

Interestingly, Medicaid acceptance was associated with much lower odds of being a nonadopter across time periods. One explanation for this may be that facilities accepting Medicaid are also likely to have different target clientele, including those with more limited time and resources who might therefore benefit uniquely from the improved accessibility through telehealth.^16^ Given the prominent role of individual states in determining Medicaid benefits, future research might evaluate the extent to which trajectories of telehealth coverage vary according to state Medicaid policies.

Our analysis benefited from a secret shopper approach, which reduces social desirability bias among respondents. We successfully contacted over 1000 MHTFs throughout the US both during and after the end of the PHE. Our analysis of nonresponse bias also suggests that the analytic sample is representative of MHTFs more generally that report to SAMHSA, although we can only state this with respect to observable characteristics.

### Limitations

This study had limitations, including potential nonresponse bias, changes in telehealth policy apart from the PHE that could influence MHTF operations, and insufficient statistical power to conduct comparative analyses across other sociodemographic categories. Appropriateness of telehealth vs in-person care may differ across heterogeneous populations, as may quality of care, and our study is unable to comment on this.

## Conclusions

This longitudinal study identified evolving patterns in telehealth availability for mental health care over a 1-year period during which the COVID-19 PHE expired. Our results emphasize the importance of monitoring access to mental health care in a postpandemic era, especially against the backdrop of the federal and state policy landscape.

## Notes

Supplement 1. **eAppendix.** Study Protocol

Supplement 2.
Data Sharing Statement

## References

[1] McBain RK, Schuler MS, Qureshi N, . Expansion of telehealth availability for mental health care after state-level policy changes from 2019 to 2022. JAMA Netw Open. 2023;6(6):e2318045. doi:10.1001/jamanetworkopen.2023.18045 37310741 PMC10265313

[2] Blanco C, Wall MM, Olfson M. Implications of telepsychiatry for cost, quality, and equity of mental health care. JAMA Psychiatry. 2022;79(12):1147-1148. doi:10.1001/jamapsychiatry.2022.3330 36260304

[3] Shachar C, Engel J, Elwyn G. Implications for telehealth in a postpandemic future: regulatory and privacy issues. JAMA. 2020;323(23):2375-2376. doi:10.1001/jama.2020.7943 32421170

[4] Chu RC, Peters C, Lew ND, Sommers BD. State Medicaid telehealth policies before and during the COVID-19 public health emergency. Office of the Assistant Secretary for Planning and Evaluation, U.S. Department of Health and Human Services. 2021. Accessed June 5, 2024. https://aspe.hhs.gov/sites/default/files/documents/eb9e147935a2663441a9488e36eea6cb/medicaid-telehealth-brief.pdf

[5] Department of Health and Human Services. Telehealth policy changes after the COVID-19 public health emergency. January 19, 2023. Accessed January 30, 2024. https://telehealth.hhs.gov/providers/telehealth-policy/policy-changes-after-the-covid-19-public-health-emergency

[6] American Academy of Family Physicians. Telehealth after the COVID-19 PHE: what’s changing, and what’s staying the same for now. 2023. Accessed May 2, 2024. https://www.aafp.org/pubs/fpm/blogs/inpractice/entry/covid-phe-end-telehealth.html#:~:text=The%20originating%20and%20geographic%20site,to%20a%20health%20care%20facility

[7] Centers for Medicare and Medicaid Services. Telehealth Services. 2024. Accessed June 5, 2024. https://www.cms.gov/files/document/mln901705-telehealth-services.pdf

[8] Substance Abuse and Mental Health Services Administration. FindTreatment.gov. 2023. Accessed June 3, 2023. https://findtreatment.gov/

[9] Substance Abuse and Mental Health Services Administration. SAMHSA behavioral health treatment services locator. 2022. Accessed May 17, 2022. https://findtreatment.samhsa.gov/locator/about.html#.YoOJ5-hlA2w

[10] Health Resources and Services Administration. Area health resources files. Accessed January 31, 2024. https://data.hrsa.gov/topics/health-workforce/ahrf

[11] Patrick SW, Richards MR, Dupont WD, . Association of pregnancy and insurance status with treatment access for opioid use disorder. JAMA Netw Open. 2020;3(8):e2013456. doi:10.1001/jamanetworkopen.2020.13456 32797175 PMC7428808

[12] Beetham T, Saloner B, Gaye M, Wakeman SE, Frank RG, Barnett ML. Therapies offered at residential addiction treatment programs in the United States. JAMA. 2020;324(8):804-806. doi:10.1001/jama.2020.8969 32840587 PMC7448823

[13] Maleka NH, Matli W. A review of telehealth during the COVID-19 emergency situation in the public health sector: challenges and opportunities. J Sci Tech Pol Manage. Published online July 29, 2022. doi:10.1108/JSTPM-08-2021-0126

[14] Phuong J, Ordóñez P, Cao J, . Telehealth and digital health innovations: a mixed landscape of access. PLOS Digit Health. 2023;2(12):e0000401. doi:10.1371/journal.pdig.0000401 38100519 PMC10723719

[15] Cantor JH, McBain RK, Kofner A, Stein BD, Yu H. Availability of outpatient telemental health services in the United States at the outset of the COVID-19 pandemic. Med Care. 2021;59(4):319-323. doi:10.1097/MLR.0000000000001512 33480660 PMC7954880

[16] Guth M. Telehealth delivery of behavioral health care in Medicaid: findings from a survey of state Medicaid programs. KFF. January 10, 2023. Accessed May 2, 2024. https://www.kff.org/mental-health/issue-brief/telehealth-delivery-of-behavioral-health-care-in-medicaid-findings-from-a-survey-of-state-medicaid-programs/

