# Supplementary material for: Telehealth Availability for Mental Health Care During and After the COVID-19 Public Health Emergency
Source: JAMA Netw Open. 2024 Jul 10;7(7):e2420853. doi: 10.1001/jamanetworkopen.2024.20853 (PMC11238022; doi:10.1001/jamanetworkopen.2024.20853)
Supplement: Supplement 1. — eAppendix. Study Protocol [file jamanetwopen-e2420853-s001.pdf]

Supplemental Online Content

McBain RK, Schuler MS, Breslau J, Kofner A, Wang L, Cantor JH. Telehealth availability for mental health care during and after the COVID-19 public health emergency. *JAMA Netw Open*. 2024;7(7):e2420853. doi:10.1001/jamanetworkopen.2024.20853

eAppendix. Study Protocol

This supplemental material has been provided by the authors to give readers additional information about their work.

© 2024 McBain RK et al. *JAMA Network Open.*

CASE 1: MDD + AUD

Background: Adult diagnosed in 2019 with major depressive disorder (MDD), who hasn't received behavioral therapy in a while, but would like to re-engage using telehealth due to feeling more depressed over the past 3 months: lack of energy, feeling hopeless, trouble sleeping, overeating, unemployed since start of COVID pandemic. They do NOT have active suicidal ideation. They are curious about receiving cognitive behavioral therapy and having their medications adjusted (they are currently taking a low dose of Prozac). They are on Medicaid but are willing to pay out of pocket if need be.

1. For Interviewer: What is the Facility ID? ____________ (validation: always 6 numeric digits)

2. For Interviewer: Hi, my name is [NAME].
   a. Lakisha Washington (Black/Female)
   b. Jamal Jones (Black/Male)
   c. Isabella Hernandez (Hispanic/Female)
   d. Carlos García (Hispanic/Male)
   e. Emily Walsh (White/Female)
   f. Greg Baker (White/Male)

3. Are you accepting new patients right now?
   a. Yes →Great [Proceed to #4]
   b. No → I see. Do you mind if I ask a few questions about your services anyway, in case you start taking new patients in the future?
      i. No→Okay, thank you. [Hang up]
      ii. Yes→Okay. [Proceed to #4]

4. Do you take Medicaid?
   a. Yes→Okay. [Skip to #6]
   b. No→I see. [Proceed to #5]
   c. I don't know→Okay. [Skip to #6]

5. Is it only out-of-pocket payment that you accept, or do you take private insurance?
   a. Out of pocket only [Proceed to #6]
   b. Private insurance AND out of pocket [Proceed to #6]
   c. Other insurance [Record] [Proceed to #6]

6. Do you provide any telehealth services?
   a. Yes→I see. [Proceed to #7]
   b. No→I see. Okay, well, thank you for your time. [Hang up]
   c. I don't know→I see. Okay, well, thank you for your time. [Hang up]

7. Do you reserve any telehealth appointment slots for same-day or next day telehealth visits, for example for folks with more urgent needs?
   a. Yes→I see. [Proceed to #8]
   b. No→I see. [Proceed to #8]
   c. I don't know→I see. [Proceed to #8]

8. Are telehealth services by phone or by video, or do you have both?

© 2024 McBain RK et al. *JAMA Network Open.*

a. Phone [Proceed to #9]
b. Video [Proceed to #9]
c. Both [Proceed to #9]
d. I don't know [Proceed #9]

9. What is the primary platform you use for telehealth appointments? E.g., Zoom, WebEx, FaceTime, Skype, Doxy
    a. [Record software]. Okay. [Proceed to #10]
    b. I don't know →Okay. [Proceed to #10]

10. Do you provide telehealth counseling / behavioral therapy for patients *with depression*?
    a. Yes → Great [Proceed to #11]
    b. Yes, but with exceptions → What exceptions? [Record response] [Proceed to #11]
    c. No → I see. What does this facility provide telehealth counseling / behavioral therapy for? [Record response] [Proceed to #11]
    d. I don't know → I see. What does this facility provide telehealth counseling / behavioral therapy for? [Record response] [Proceed to #11]

11. Do you provide medication management through telehealth for patients *with depression* (I am currently taking *Prozac*)?
    a. Yes → Great [Proceed to #12].
    b. Yes, but with exceptions → What exceptions? [Record response] [Proceed to #12]
    c. No → I see. What does this facility provide medication management for? [Record response] [Proceed to #12]
    d. I don't know → I see. What does this facility provide t medication management for? [Record response] [Proceed to #12]

12. Does this facility provide diagnostic services using telehealth? I have been previously diagnosed with *depression*, but it was several years ago.
    a. Yes → Great [Proceed to #13].
    b. Yes, but with exceptions → What exceptions? [Record response] [Proceed to #13]
    c. No → I see. What does this facility provide diagnostic services for? [Record response] [Proceed to #13]
    d. I don't know → I see. What does this facility provide diagnostic services for? [Record response] [Proceed to #13]

Coding Note: If response to 10, 11, and 12 are all "No", SKIP to Question 14 (because the facility doesn't offer any telehealth services for the mental health condition in question).

13. What's the soonest date that you could schedule me for a telehealth appointment?
    a. [Record response]. Okay, gotcha. [Proceed to #14]
    b. I can't give this to you right now → Okay. [Proceed to #14]

14. Also, do you provide telehealth counseling / behavioral therapy for people who want to work on their *drinking*?
    a. Yes→Great [Proceed to #15]
    b. No→I see. [Proceed to #15]
    c. I don't know→I see. [Proceed to #15]

© 2024 McBain RK et al. *JAMA Network Open.*

15. Does this facility provide medication treatment for *alcohol problems* through telehealth?
    a. Yes→Okay. [Proceed to #16]
    b. No→ Okay. [Proceed to #16]
    c. I don't know→ Okay. [Proceed to #16]

16. Does this facility provide diagnostic services for *alcohol problems* through telehealth? I'm not sure if I really have a problem with *alcohol* or if I'm just *depressed*.
    a. Yes→Okay. [Proceed to #17]
    b. No→ Okay. [Proceed to #17]
    c. I don't know→ Okay. [Proceed to #17]

17. Would the same treatment provider be able to work with me on both my *depression and alcohol*, or would I have to see different providers?
    a. Concurrent treatment with one provider →I see. [Proceed to #18]
    b. Two separate providers →I see. [Proceed to #18]
    c. Sometimes the same, sometimes different → I see. [Proceed to #18]
    d. I don't know→Okay. [Proceed to #18]

18. Can I text providers in-between appointments with questions/concerns?
    a. Yes→I see. [Proceed to #19]
    b. No→I see. [Proceed to #19]
    c. I don't know→I see. [Proceed to #19]

19. One last question: do you offer telehealth services to patients residing in other states?
    a. Yes → Okay. Before I move forward with this, I want to take some time to think about your answers, but I really appreciate your time! [Hang up]
    b. No → Okay. Before I move forward with this, I want to take some time to think about your answers, but I really appreciate your time! [Hang up]
    c. I don't know → Okay. Before I move forward with this, I want to take some time to think about your answers, but I really appreciate your time! [Hang up]
    d. Which state are you in? [Provide a response that is a neighboring state]. Before I move forward with this, I want to take some time to think about your answers, but I really appreciate your time! [Hang up]

20. If you have any qualitative notes you want to document from your conversation, please list here.

[SUBMIT]

© 2024 McBain RK et al. *JAMA Network Open.*

CASE 2: GAD + AUD, LOOKING TO RE-ENGAGE IN CBT/ADT

Background: Adult diagnosed in 2019 with generalized anxiety disorder (GAD), who hasn't received behavioral therapy in a while, but would like to re-engage using telehealth due to feeling more anxious over the past 3 months: unexplained panic attacks that are accompanied by physical distress—including restlessness, feeling on edge, difficulty concentrating, muscle tension, trouble falling asleep, teeth clenching. They have been feeling low energy/down, but they attribute it to the impairment from their anxiety. They are curious about receiving cognitive behavioral therapy and having their medications adjusted (they are currently taking a low dose of Lexapro). They are on Medicaid but are willing to pay out of pocket if need be.

1. For Interviewer: What is the Facility ID? ____________ (validation: always 6 numeric digits)

2. For Interviewer: Hi, my name is [NAME].
   a. Lakisha Washington (Black/Female)
   b. Jamal Jones (Black/Male)
   c. Isabella Hernandez (Hispanic/Female)
   d. Carlos García (Hispanic/Male)
   e. Emily Walsh (White/Female)
   f. Greg Baker (White/Male)

3. Are you accepting new patients right now?
   a. Yes →Great [Proceed to #4]
   b. No → I see. Do you mind if I ask a few questions about your services anyway, in case you start taking new patients in the future?
      i. No→Okay. [Hang up]
      ii. Yes→Okay. [Proceed to #4]

4. Do you take Medicaid?
   a. Yes→Okay. [Skip to #6]
   b. No→I see. [Proceed to #5]
   c. I don't know→Okay. [Skip to #6]

5. Is it only out-of-pocket payment that you accept, or do you take private insurance?
   a. Out of pocket only [Proceed to #6]
   b. Private insurance AND out of pocket [Proceed to #6]
   c. Other insurance [Record] [Proceed to #6]

6. Do you provide any telehealth services?
   a. Yes→I see. [Proceed to #7]
   b. No→I see. Okay, well, thank you for your time. [Hang up]
   c. I don't know→I see. Okay, well, thank you for your time. [Hang up]

© 2024 McBain RK et al. *JAMA Network Open.*

7. Do you reserve any telehealth appointment slots for same-day or next day telehealth visits, for example for folks with more urgent needs?
    a. Yes→I see. [Proceed to #8]
    b. No→I see. [Proceed to #8]
    c. I don't know→I see. [Proceed to #8]

8. Are telehealth services by phone or by video, or do you have both?
    a. Phone [Proceed to #9]
    b. Video [Proceed to #9]
    c. Both [Proceed to #9]
    d. I don't know [Proceed #9]

9. What is the primary platform you use for telehealth appointments? E.g., Zoom, WebEx, FaceTime, Skype, Doxy
    a. [Record software]. Okay. [Proceed to #10]
    b. I don't know →Okay. [Proceed to #10]

10. Do you provide telehealth counseling / behavioral therapy for patients *with anxiety*?
    a. Yes → Great [Proceed to #11]
    b. Yes, but with exceptions → What exceptions? [Record response] [Proceed to #11]
    c. No → I see. What does this facility provide telehealth counseling / behavioral therapy for? [Record response] [Proceed to #11]
    d. I don't know → I see. What does this facility provide telehealth counseling / behavioral therapy for? [Record response] [Proceed to #11]

11. Do you provide medication management through telehealth for patients *with anxiety* (I am currently taking *Lexapro*)?
    a. Yes → Great [Proceed to #12].
    b. Yes, but with exceptions → What exceptions? [Record response] [Proceed to #12]
    c. No → I see. What does this facility provide medication management for? [Record response] [Proceed to #12]
    d. I don't know → I see. What does this facility provide t medication management for? [Record response] [Proceed to #12]

12. Does this facility provide diagnostic services using telehealth? I have been previously diagnosed with *generalized anxiety disorder*, but it was several years ago.
    a. Yes → Great [Proceed to #13].
    b. Yes, but with exceptions → What exceptions? [Record response] [Proceed to #13]
    c. No → I see. What does this facility provide diagnostic services for? [Record response] [Proceed to #13]
    d. I don't know → I see. What does this facility provide diagnostic services for? [Record response] [Proceed to #13]

© 2024 McBain RK et al. *JAMA Network Open.*

Coding Note: If response to 10, 11, and 12 are all "No", the caller should thank the provider for their time and then SKIP to Question 14 (because the facility doesn't offer any telehealth services for the mental health condition in question).

13. What's the soonest date that you could schedule me for a telehealth appointment?
    a. [Record response]. Okay, gotcha. [Proceed to #14]
    b. I can't give this to you right now → Okay. [Proceed to #14]

14. Also, do you provide telehealth counseling / behavioral therapy for people who want to work on their *drinking*?
    d. Yes→Great [Proceed to #15]
    e. No→I see. [Proceed to #15]
    f. I don't know→I see. [Proceed to #15]

15. Does this facility provide medication treatment for *alcohol problems* through telehealth?
    a. Yes→Okay. [Proceed to #16]
    b. No→ Okay. [Proceed to #16]
    c. I don't know→ Okay. [Proceed to #16]

16. Does this facility provide diagnostic services for *alcohol problems* through telehealth? I'm not sure if I really have a problem with *alcohol* or if I'm just *anxious*.
    a. Yes→Okay. [Proceed to #17]
    b. No→ Okay. [Proceed to #17]
    c. I don't know→ Okay. [Proceed to #17]

17. Would the same treatment provider be able to work with me on both my *anxiety and alcohol use*, or would I have to see different providers?
    a. Concurrent treatment with one provider →I see. [Proceed to #18]
    b. Two separate providers →I see. [Proceed to #18]
    c. Sometimes the same, sometimes different → I see. [Proceed to #18]
    d. I don't know→Okay. [Proceed to #18]

18. Can I text providers in-between appointments with questions/concerns?
    a. Yes→I see. [Proceed to #19]
    b. No→I see. [Proceed to #19]
    c. I don't know→I see. [Proceed to #19]

19. One last question: do you offer telehealth services to patients residing in other states?
    e. Yes → Okay. Before I move forward with this, I want to take some time to think about your answers, but I really appreciate your time! [Hang up]
    f. No → Okay. Before I move forward with this, I want to take some time to think about your answers, but I really appreciate your time! [Hang up]
    g. I don't know → Okay. Before I move forward with this, I want to take some time to think about your answers, but I really appreciate your time! [Hang up]
    h. Which state are you in? [Provide a response that is a neighboring state]. Before I move forward with this, I want to take some time to think about your answers, but I really appreciate your time! [Hang up]

© 2024 McBain RK et al. *JAMA Network Open.*

20. If you have any qualitative notes you want to document from your conversation, please list here.

[SUBMIT]

CASE 3: SZ + AUD, LOOKING TO RE-ENGAGE IN CBT/ADT

Background: Adult diagnosed in 2019 with schizophrenia (SZ), who hasn't received counseling/behavioral therapy in a while, but would like to re-engage using telehealth. Their symptom profile includes: paranoia (particularly that friends/family members are 'out to get' them), trouble keeping their thoughts straight, difficulties with self-care, social isolation. When they aren't on their meds (which they currently are), they have occasionally heard voices that other people haven't. They are curious about receiving cognitive behavioral therapy and having their medications adjusted (they are currently taking Risperdal at 4 mg/day). They are on Medicaid but are willing to pay out of pocket if need be.

1. For Interviewer: What is the Facility ID? ____________ (validation: always 6 numeric digits)

2. For Interviewer: Hi, my name is [NAME].
   a. Lakisha Washington (Black/Female)
   b. Jamal Jones (Black/Male)
   c. Isabella Hernandez (Hispanic/Female)
   d. Carlos García (Hispanic/Male)
   e. Emily Walsh (White/Female)
   f. Greg Baker (White/Male)

3. Are you accepting new patients right now?
   a. Yes →Great [Proceed to #4]
   b. No → I see. Do you mind if I ask a few questions about your services anyway, in case you start taking new patients in the future?
      i. No→Okay. [Hang up]
      ii. Yes→Okay. [Proceed to #4]

4. Do you take Medicaid?
   a. Yes→Okay. [Skip to #6]
   b. No→I see. [Proceed to #5]
   c. I don't know→Okay. [Skip to #6]

5. Is it only out-of-pocket payment that you accept, or do you take private insurance?
   a. Out of pocket only [Proceed to #6]
   b. Private insurance AND out of pocket [Proceed to #6]
   c. Other insurance [Record] [Proceed to #6]

6. Do you provide any telehealth services?
   a. Yes→I see. [Proceed to #7]
   b. No→I see. Okay, well, thank you for your time. [Hang up]
   c. I don't know→I see. Okay, well, thank you for your time. [Hang up]

7. Do you reserve any telehealth appointment slots for same-day or next day telehealth visits, for example for folks with more urgent needs?
   a. Yes→I see. [Proceed to #8]
   b. No→I see. [Proceed to #8]
   c. I don't know→I see. [Proceed to #8]

8. Are telehealth services by phone or by video, or do you have both?
    a. Phone [Proceed to #8]
    b. Video [Proceed to #8]
    c. Both [Proceed to #8]
    d. I don't know [Proceed #8]

9. What is the primary platform you use for telehealth appointments? E.g., Zoom, WebEx, FaceTime, Skype, Doxy
    a. [Record software]. Okay. [Proceed to #10]
    b. I don't know →Okay. [Proceed to #10]

10. Do you provide telehealth counseling / behavioral therapy for patients *with schizophrenia*?
    a. Yes → Great [Proceed to #11]
    b. Yes, but with exceptions → What exceptions? [Record response] [Proceed to #11]
    c. No → I see. What does this facility provide telehealth counseling / behavioral therapy for? [Record response] [Proceed to #11]
    d. I don't know → I see. What does this facility provide telehealth counseling / behavioral therapy for? [Record response] [Proceed to #11]

11. Do you provide medication management through telehealth for patients *with schizophrenia* (I am currently taking *Risperidone*)?
    a. Yes → Great [Proceed to #12].
    b. Yes, but with exceptions → What exceptions? [Record response] [Proceed to #12]
    c. No → I see. What does this facility provide medication management for? [Record response] [Proceed to #12]
    d. I don't know → I see. What does this facility provide t medication management for? [Record response] [Proceed to #12]

12. Does this facility provide diagnostic services using telehealth? I have been previously diagnosed with *schizophrenia*, but it was several years ago.
    a. Yes → Great [Proceed to #13].
    b. Yes, but with exceptions → What exceptions? [Record response] [Proceed to #13]
    c. No → I see. What does this facility provide diagnostic services for? [Record response] [Proceed to #13]
    d. I don't know → I see. What does this facility provide diagnostic services for? [Record response] [Proceed to #13]

Coding Note: If response to 10, 11, and 12 are all "No", the caller should thank the provider for their time and then SKIP to Question 14 (because the facility doesn't offer any telehealth services for the mental health condition in question).

13. What's the soonest date that you could schedule me for a telehealth appointment?
    a. [Record response]. Okay, gotcha. [Proceed to #14]
    b. I can't give this to you right now → Okay. [Proceed to #14]

14. Also, do you provide telehealth counseling / behavioral therapy for people who want to work on their *drinking*?
    g. Yes→Great [Proceed to #15]

© 2024 McBain RK et al. *JAMA Network Open.*

h. No→I see. [Proceed to #15]
i. I don't know→I see. [Proceed to #15]

15. Does this facility provide medication treatment for *alcohol problems* through telehealth?
    a. Yes→Okay. [Proceed to #16]
    b. No→ Okay. [Proceed to #16]
    c. I don't know→ Okay. [Proceed to #16]

16. Does this facility provide diagnostic services for *alcohol problems* through telehealth? I'm not sure if I really have a problem with *alcohol* or if it's just my *schizophrenia*.
    a. Yes→Okay. [Proceed to #17]
    b. No→ Okay. [Proceed to #17]
    c. I don't know→ Okay. [Proceed to #17]

17. Would the same treatment provider be able to work with me on both my *schizophrenia and alcohol use*, or would I have to see different providers?
    a. Concurrent treatment with one provider →I see. [Proceed to #18]
    b. Two separate providers →I see. [Proceed to #18]
    c. Sometimes the same, sometimes different → I see. [Proceed to #18]
    d. I don't know→Okay. [Proceed to #18]

18. Can I text providers in-between appointments with questions/concerns?
    c. Yes→I see. [Proceed to #19]
    d. No→I see. [Proceed to #19]
    e. I don't know→I see. [Proceed to #19]

19. One last question: do you offer telehealth services to patients residing in other states?
    i. Yes → Okay. Before I move forward with this, I want to take some time to think about your answers, but I really appreciate your time! [Hang up]
    j. No → Okay. Before I move forward with this, I want to take some time to think about your answers, but I really appreciate your time! [Hang up]
    k. I don't know → Okay. Before I move forward with this, I want to take some time to think about your answers, but I really appreciate your time! [Hang up]
    l. Which state are you in? [Provide a response that is a neighboring state]. Before I move forward with this, I want to take some time to think about your answers, but I really appreciate your time! [Hang up]

20. If you have any qualitative notes you want to document from your conversation, please list here.

[SUBMIT]

© 2024 McBain RK et al. *JAMA Network Open.*
