# Supplementary material for: Telehealth Availability for Mental Health Care During and After the COVID-19 Public Health Emergency
Source: JAMA Netw Open. 2024 Jul 10;7(7):e2420853. doi: 10.1001/jamanetworkopen.2024.20853 (PMC11238022; doi:10.1001/jamanetworkopen.2024.20853)
Supplement: Supplement 2. — Data Sharing Statement [file jamanetwopen-e2420853-s002.pdf]

Data Sharing Statement

McBain. Telehealth Availability for Mental Health Care During and After the COVID-19 Public Health Emergency. *JAMA Netw Open*. Published July 10, 2024. doi:10.1001/jamanetworkopen.2024.20853

Data
Data available: Yes
Data types: Deidentified participant data
How to access data: Data provided by study lead author, upon request
When available: With publication

Supporting Documents
Document types: Statistical/analytic code
How to access documents: Data provided by study lead author, upon request
When available: With publication

Additional Information
Who can access the data: Researchers who proposed use of the data has been approved
Types of analyses: For research purposes
Mechanisms of data availability: After approval of proposal
Any additional restrictions: NA
